# Pneumothorax as a Complication of Bevacizumab-Containing Chemotherapy: A Systematic Review of Case Reports

**DOI:** 10.7759/cureus.27338

**Published:** 2022-07-27

**Authors:** Shafi Rehman, Hameed Ullah, Jai Sivanandan Nagarajan, Mahnoor Sukaina, Bushra Ghafoor, Shameera Shaik Masthan, Shazmah Shahrukh, Hassan Min Allah, Muhammad Hamza Qureshi

**Affiliations:** 1 Internal Medicine, Lady Reading Hospital, Peshawar, PAK; 2 Internal Medicine, Hayatabad Medical Complex, Peshawar, Peshawar, PAK; 3 Internal Medicine, SRM Medical College Hospital and Research Centre, Chennai, IND; 4 Medicine, Karachi Medical and Dental College, Karachi, PAK; 5 Research, University of Texas Southwestern Medical Center, Dallas, USA; 6 Graduate Medical Education, Dera Ghazi Khan Medical College, Dera Ghazi Khan, PAK; 7 Internal Medicine, Nishtar Medical University, Multan, PAK; 8 Medicine, University of Louisville, Kentucky, USA; 9 Internal Medicine, Dow University of Health Sciences, Civil Hospital Karachi, Karachi, PAK; 10 Internal Medicine, Multan Medical and Dental College, Multan, PAK; 11 Internal Medicine, Mayo Hospital, Lahore, PAK

**Keywords:** rare side effect, chemotherapy-related toxicity, avastin, bevacizumab toxicity, pneumothorax (ptx)

## Abstract

Bevacizumab is a monoclonal anti-vascular endothelial growth factor (VEGF) antibody that binds to and makes all of the VEGF isoforms inactive, and thus prevents angiogenesis, development, and the spread of the tumor. The most reported side effects after administering bevacizumab include bleeding, high blood pressure, heart failure, proteinuria, thrombosis, and gastrointestinal perforation. Pneumothorax has rarely been reported as a complication of bevacizumab, but with an unclear mechanism. This article aims to explore the occurrence of pneumothorax as a side effect after using bevacizumab through a systematic review of current case reports published on the topic. A literature search was conducted using PubMed, Google Scholar, ScienceDirect, and Directory of Open Access through the utilization of appropriate keywords, and case reports were selected based on predefined inclusion and exclusion criteria. Our results encompass five case reports that were further evaluated for demographic, clinical, and treatment parameters. This systematic review concludes that pneumothorax can occur after bevacizumab-containing chemotherapy although this side effect is relatively rare. Awareness regarding this possible side effect can assist clinicians during their practice in considering pneumothorax as a possible differential diagnosis when encountering patients presenting with pulmonary symptoms after starting bevacizumab-containing chemotherapy; hence, timely diagnosis and treatment can save a life.

## Introduction and background

Bevacizumab, a monoclonal anti-vascular endothelial growth factor (VEGF) antibody, binds to and inactivates all VEGF isoforms to prevent angiogenesis, development, and spreading of the tumor [1-4]. Additionally, it restores normal tumor blood flow by inhibiting the production of vasodilator mediators [5]. Malignant tumor development and survival are greatly influenced by angiogenesis. Bevacizumab has become a mainstay in several combination chemotherapy regimens to treat patients with metastatic colorectal cancer (mCRC), metastatic breast cancer (MBC), metastatic non-small-cell lung cancer (NSCLC), metastatic renal cell carcinoma (RCC), and glioblastoma multiforme (GBM). It has a statistically significant positive impact on overall survival (OS) and progression-free survival (PFS) according to several clinical studies [3]. Bevacizumab has a substantial number of side effects, including bleeding, high blood pressure, heart failure, proteinuria, thrombosis, and gastrointestinal (GI) perforation [6]. Pneumothorax has rarely been reported as a complication of bevacizumab [7-13]. The mechanism underlying the development of pneumothorax after bevacizumab therapy is not clear. In a period where VEGF inhibitors are being used in the treatment of numerous cancers, we consider the current literature assessment of all case reports as it provides information on a rare adverse event associated with the use of bevacizumab. Pneumothorax is a possible adverse event that clinicians should be aware of to have a high clinical index of suspicion while establishing the diagnosis.

## Review

Methodology

We entered our proposed idea of the systematic review in the PROSPERO online registry on May 23, 2022, and got registered on June 3, 2022 (PROSPERO identifier: CRD42022334585). To explore our research question, we looked into the following databases: PubMed, Google Scholar, ScienceDirect, and Directory of Open Access Journal. We collected all relevant reports electronically upon entering the relevant keywords described below without using an automated tool. The cut-off date for searching the databases was June 10, 2022. We applied a Boolean scheme to the keywords while incorporating the Medical Subject Heading (MeSH) strategy. The articles were checked for titles/abstracts while setting inclusion/exclusion criteria which are mentioned below. We restricted to the Preferred Reporting Items for Systematic Reviews and Meta-Analyses (PRISMA) guidelines 2020 in this systemic review [14].

Inclusion and Exclusion Criteria

We included only case reports published in the English language with free open access to full-text reports across the globe with no time restriction including humans as subjects only. All other study designs were excluded, including non-English, and non-full text articles and articles for which we had to pay for a subscription. All included studies fulfilled the quality assessment.

Keywords

MeSH keywords searched in PubMed included: Pneumothorax OR Spontaneous Pneumothorax OR Tension Pneumothorax OR Pressure Pneumothorax OR Primary Spontaneous Pneumothorax OR Secondary Spontaneous Pneumothorax OR (“Pneumothorax/anatomy and histology”[Mesh] OR “Pneumothorax/diagnosis”[Mesh] OR “Pneumothorax/diagnostic imaging”[Mesh] OR “Pneumothorax/drug therapy”[Mesh]) AND Bevacizumab OR Avastin OR (“Bevacizumab/adverse effects”[Mesh] OR “Bevacizumab/drug effects”[Mesh] OR “Bevacizumab/toxicity”[Mesh]).

Keywords searched in other databases included pneumothorax, spontaneous pneumothorax, tension pneumothorax, pressure pneumothorax, primary spontaneous pneumothorax, secondary spontaneous pneumothorax, bevacizumab, and Avastin.

Quality assessment

During the selection of the case reports, we used a quality appraisal tool, the Joanna Briggs Institute (JBI) Critical Appraisal Checklist for Case Reports, and two researchers worked independently on data selection and extraction. In situations where we could not agree, we discussed the study designs, inclusion and exclusion criteria, intervention employed, and results. When we encountered any difficulty in the selection of studies, we approached the third reviewer to help resolve disagreements and find common ground. The quality of the selected case reports is depicted in Table 1.

**Table 1:** Quality assessment of case reports. 1. Were the patient’s demographic characteristics clearly described? 2. Was the patient’s history clearly described and presented as a timeline? 3. Was the current clinical condition of the patient on presentation clearly described? 4. Were diagnostic tests or assessment methods and results clearly described? 5. Was the intervention or treatment procedure clearly described? 6. Was the post-intervention clinical condition clearly described? 7. Was the adverse events or unanticipated events identified and described? 8. Does the case report provide takeaway lessons? JBI: Joanna Briggs Institute

1	2	3	4	5	6	7	8	Outcomes	Authors
Yes	Yes	Yes	Yes	Yes	No	No	Yes	Yes	Lida et al. [11]
Yes	Yes	Yes	Yes	Yes	Yes	No	Yes	Yes	Yang et al. [8]
Yes	Yes	Yes	Yes	Yes	Yes	No	Yes	Yes	Zhang et al. [3]
Yes	Yes	Yes	Yes	Yes	Yes	No	Yes	Yes	Alrifai et al. [15]
Yes	Yes	Yes	Yes	Yes	Yes	No	Yes	Yes	Ozaki et al. [16]

Results

**Figure 1:**
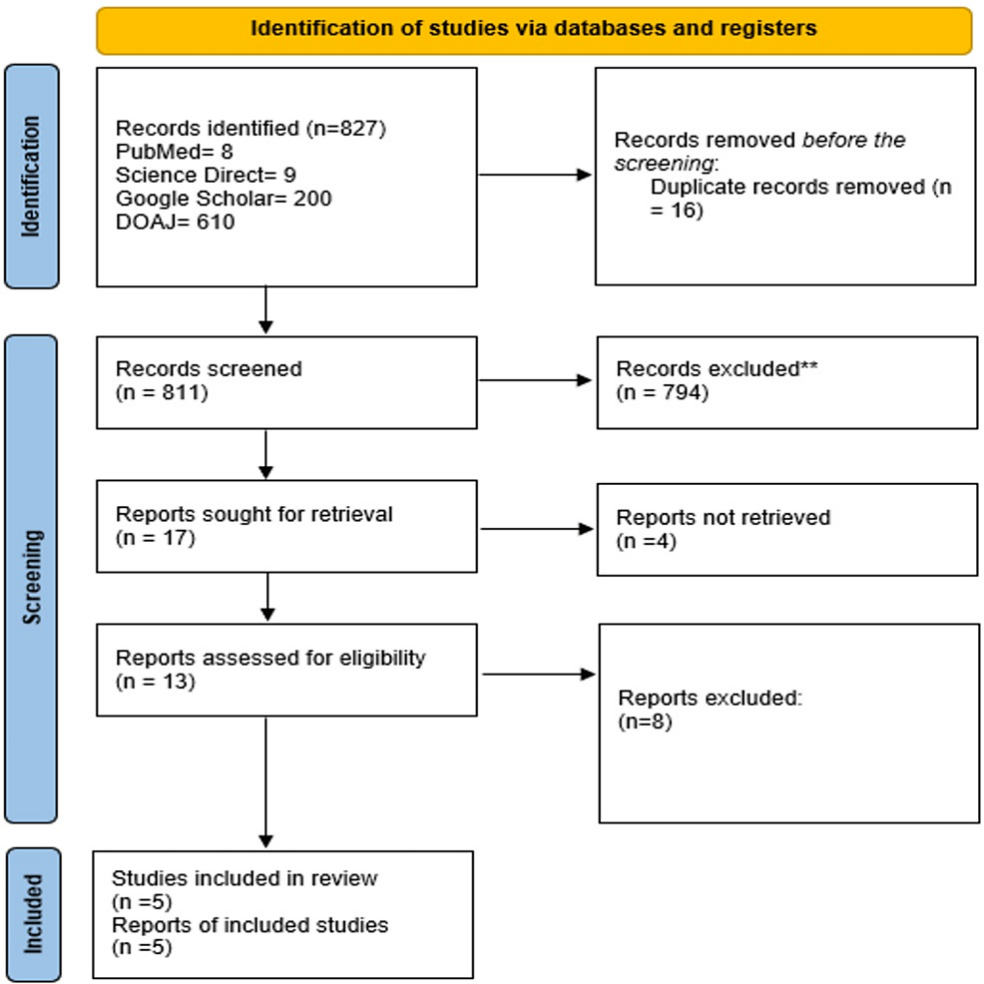
Search results depicted in the PRISMA flowchart 2020. PRISMA: Preferred Reporting Items for Systematic Reviews and Meta-Analyses

The search strategy used in this review included the above-mentioned databases and yielded 827 articles, out of which 16 were removed as they were duplicates using Zotero. A total of 811 records were screened, out of which 794 were excluded based on the relevance and the inclusion/exclusion criteria. Four reports could not be retrieved. The final screening resulted in 13 reports to check for quality and eligibility. Finally, five studies were included in this review. The PRISMA flow diagram is depicted in Figure 1 [14].

The demographics and highlights of the case reports are discussed in Table 2 below.

**Table 2:** Highlights of the included case reports. CT: computed tomography; PET/CT: positron emission tomography/computed tomography

Study (Author)	Gender	Age in years	Ethnicity	Clinical presentation	Duration of symptoms	Diagnostic tools used (CT, MRI, Biopsy, etc.)	Intervention and dosage	Results (tumor markers, biopsy reports, etc.) and the highlight of the case reports	Confirmed diagnosis
Lida et al. [11]	Male	57	Not mentioned	Pain on the left side of the chest	N/A	Endoscopy, biopsy, CT	Primary therapy: bevacizumab dose: 7.5 mg/kg on day one with XELOX (capecitabine, oxaliplatin). Secondary therapy: bevacizumab dose: 7.5 mg/kg on day one with FOLFIRI [irinotecan (CPT-11), l-leucovorin, and 5-fluorouracil (5-FU)] therapy	The endoscopy of the lower GI tract reported a tumor in the sigmoid colon. The biopsy report elucidated well-differentiated adenocarcinoma while reports of CT scan demonstrated bilateral metastasis of the lungs. No KRAS mutations were found, while colon carcinoma was IV A (UICC) staged and according to reports found to be pT3N2bM1a. After the administration of drug intervention, the metastatic lesion progressed. Secondary therapy was started to curb the effects. The patient complained of pain on the left side of the thoracic cavity. CT scan elucidated cavities and deeply infiltrated bullae in lung fields S1, S2, and continued to S4 which broadened to the pleural cavity. On the present day, the metastatic lesion ruptured causing bleeding. Two days later, shortness of breathing was reported which was diagnosed to be due to pneumothorax due to the construction of a check valve	Bevacizumab is significantly associated with pneumothorax
Yang et al. [8]	Male	45	N/A	Sudden onset of chest pain and dyspnea	Seven days after the second cycle of bevacizumab and *FOLFOXIRI* (21 days after initiation of chemotherapy)	Chest radiography, colonoscopy, biopsy, CT	Bevacizumab (5 mg/kg) plus FOLFOXIRI (oxaplatin 100 mg/m^2^, irinotecan 100 mg/m^2^, infusional 5-flurouracil/leucovorin) twice a week	1. Patient’s colonoscopy revealed a tumor measuring 20 cm from the anal verge, and the biopsy disclosed it was an adenocarcinoma. CT scan further revealed that it had metastasized to the lungs and liver. Alongside there was a partial bowel obstruction. 2. Bevacizumab (5 mg/kg) plus FOLFOXIRI (oxaplatin 100 mg/m^2^, irinotecan 100 mg/m^2^, infusional 5-fluorouracil/leucovorin) were started twice a week as first-line chemotherapy. 3. On the 21^st^ day after initiation of chemotherapy, there was a sudden onset of chest pain and dyspnea in the patient. 4. There were decreased breath sounds on the right side of the chest upon physical examination. The chest radiograph revealed a pneumothorax that was absent in the previous chest radiograph done. 5. Pneumothorax was resolved completely by chest tube insertion which remained inserted for 7 days. Follow up chest radiograph did not show pneumothorax	Bevacizumab chemotherapy is strongly associated with pneumothorax
Zhang et al. [3]	Male	23	N/A	Sudden onset of chest pain and dyspnea	13 days after the third cycle of bevacizumab plus DP (day 55 after initial chemotherapy)	Right inguinal lymph node biopsy	Bevacizumab (5 mg/kg) and DP (docetaxel 75 mg/m^2^, cisplatin 75 mg/m^2^) every 21days	The pneumothorax resolved ultimately after chest tube drainage. The chest tube was removed 7 days later, and the follow-up radiograph did not show a recurrence of the pneumothorax	The breath sounds were decreased upon the physical examination of the chest . Compared with the previous chest radiograph, a bilateral pneumothorax was disclosed
Alrifai et al. [15]	Female	68	Not mentioned	Shortness of breath, right-sided pleuritic chest pain, and a dry cough	She had experienced shortness of breath for a month. The presentation started after taking a combination of FOLFOX and bevacizumab for metastatic colorectal cancer	PET/CT scan, CT abdomen, biopsy, CEA levels, and chest- X-ray	She was initially staged as 3B metastatic colorectal cancer. Sigmoidectomy was performed and was given chemotherapy for 12 cycles of FOLFOX. Later Cyber-knife was used followed by 12 cycles of a regimen including leucovorin, 5-FU (fluorouracil), and irinotecan (FOLFIRI) with cetuximab. Cetuximab was later used as maintenance therapy. Radiofrequency ablation was used to treat Adrenal mass and chemotherapy used after that was FOLFIRI and bevacizumab and then only capecitabine. The combination of FOLFIRI and panitumumab was used for the recurrence of lesions in the right lung base. For the management of the pneumothorax, the patient underwent placement of a chest tube,10 French in size. Repeat chest X‑ray was normal	1. PET/CT scan revealed the recurrence of the disease with three metastatic lung lesions She was initially diagnosed with metastatic colorectal carcinoma for which she had undergone sigmoidectomy. 2. New onset of adrenal mass was noted on CT abdomen. 3. Metastatic adenocarcinoma was revealed on biopsy. 4. Three months later, the disease recurrence was observed after an increase in CEA levels, and PET/CT scan revealed lesions in the base of the right lung along with mediastinal lymph nodes. A mass was noted in the left adrenal. 5. Hydropneumothorax was revealed on the chest X-ray	Hydropneumothorax was observed on chest X-ray after taking FOLFOX and bevacizumab for metastatic colorectal cancer
Ozaki et al. [16]	Female	45	N/A	Dyspnea	N/A	Mammogram, core needle biopsy, chest X-ray, and CT chest	Primary treatment: 5-fluorouracil + epirubicin + cyclophosphamide (FEC) followed by paclitaxel (PTX) per week. This treatment was stopped because of tumor evolution. Postoperative chemotherapy: The patient was given 2 cycles of FEC followed by 8 cycles of capecitabine, but metastases in the lung were detected after the fifth of capecitabine bevacizumab + paclitaxel therapy was started. The length of the cycle was 28 days. The dose of bevacizumab was 10 mg/kg on the first and eighth days. The dose of paclitaxel was 80 mg/m^2^ administered on the first, eighth, and fifteenth day until dyspnea was observed on the start of cycle. Treatment of pneumothorax: Intercoastal catheter was used to drain the chest cavity. The patient was discharged on the seventh day after an X-ray showed no abnormalities. Regimen post-discharge: irinotecan, gemcitabine + carboplatin	She reported to the hospital after her mammogram was found to have some abnormalities. The cancer was detected in her left breast and she was staged as (cStageT3N1M0). The results of the core needle biopsy sample showed a triple-negative subtype of cancer. She was later diagnosed as having lung metastases bilaterally, and after starting bevacizumab developed dyspnea on physical examination, tachypnea was noticed	Pneumothorax was diagnosed on the right side following a chest X-ray. CT showed a bronchopleural fistula that led to the formation of the pneumothorax After 23 months of diagnosing breast cancer and nine months after diagnosing pneumothorax, the patient passed away

Discussion

To the best of our knowledge, this is the first systematic review of case reports supporting evidence of the association between bevacizumab and pneumothorax. The above results demonstrate that bevacizumab-associated pneumothorax is commonly found at the median age of 47.6 and is more common in males.

Pneumothorax as a side effect of bevacizumab-containing chemotherapy is not widely acknowledged. However, as mentioned above, several case reports link pneumothorax as a possible side effect of bevacizumab-containing chemotherapy. Yamada et al. classified several causes of pneumothorax during chemotherapy: (1) Accidental rupture of bullae or blebs under the pleura when undergoing chemotherapy sessions for cancer. (2) The formation of bronchopleural fistulas due to tumor necrosis. (3) The damage caused to the lung parenchyma due to chemotherapy or radiation therapy and hence the formation of pleural lesions. (4) The formation of cavities or emphysematous lesions in the peripheral tissues and rupture by the check-valve mechanism because of tumor-related obstruction or stenosis of bronchi. (5) The elevation of intrathoracic pressure due to vomiting induced by chemotherapy can cause rupture of the pleura [17].

It is difficult to determine the exact frequency of pneumothorax associated with bevacizumab, given the dearth of reported cases and the inability to determine the number of patients receiving bevacizumab therapy annually. Interiano et al. performed a retrospective analysis of pediatric patients with recurrent or refractory solid malignancies who underwent combination chemotherapy including bevacizumab. The primary goal of their analysis was to assess the risk of developing pneumothorax as a possible complication of bevacizumab-containing chemotherapy. The study reported a high incidence of pneumothorax in 11 of the 44 (25%) patients, which was unexpected [18]. Although the study was conducted in the pediatric population, its results may suggest that bevacizumab-associated pneumothorax may be happening at much higher rates than reported compared to healthy people. In comparison, primary spontaneous pneumothorax (PSP) in otherwise healthy people is estimated to be 7.4-18 cases per 100,000 among men and 6 cases per 100,000 among women. In addition, PSP rarely occurs after the age of 40 [19].

Routine treatment with bevacizumab leads to tumor necrosis. In lung metastases, for example, in sarcoma, tumor necrosis occurs due to chemotherapy and leads to internal cavitation, which results in secondary spontaneous pneumothorax in most patients [8,20]. Secondary spontaneous pneumothorax after cytotoxic chemotherapy is a rare but documented occurrence associated with primary or metastatic lung lesions [21]. Yang et al. reported that the incidence of pneumothorax after bevacizumab-containing chemotherapy in patients with colorectal cancer at their hospital was 0.51%, that is, one patient of the 193 patients treated with bevacizumab. The worldwide incidence is expected to be lower than the actual incidence [8]. However, some patients with sarcoma had bilateral spontaneous pneumothorax during chemotherapy, but pulmonary metastases were not detectable. This suggests that spontaneous pneumothorax was either a coincidence or a complication of very small metastases adjacent to the pleura [22,23]. Early detection of lung metastases is difficult. Furrer et al. suggested that imaging studies such as chest radiography and CT are suboptimal for detecting micro-lesions and that sometimes pneumothorax can be the first and only evidence for metastases [24].

Bevacizumab is used most commonly for the treatment of lung cancer, breast cancer, and colon cancer. The common side effects of bevacizumab are hypertension, proteinuria, and delayed wound healing; however, bleeding, especially GI bleeding, has been reported as a potential side effect [25-27]. Bevacizumab-induced bleeding is due to damage to vascular endothelial cells due to inhibition of angiogenesis and abnormal coagulation profile due to depletion of VEGF in platelets, which promotes the formation of thrombi [28]. Epistaxis and alveolar hemorrhage have been reported to be caused by bevacizumab [29,30]. In most patients, bleeding is light, with severe bleeding only occurring in 1.37% of patients. However, a meta-analysis tells us that bleeding is the most common cause of death related to bevacizumab therapy in patients undergoing treatment for lung cancer [31]. Bevacizumab binds to VEGF-A and inhibits its binding to VEGF receptor (VEGFR)-1 and (VEGFR)-2 and halts the VEGF signal transduction. Bevacizumab performs its antitumor effect by causing ischemic alterations, such as microvascular involution in tumors and inhibition of tumor angiogenesis. It is also known to improve drug delivery by normalizing the vascular plexus in tumor cells [32-34]. Hence, it is conceivable that the anti-angiogenic effect of bevacizumab, which leads to distortion of the tumor vasculature, may result in pneumothorax in tumors that are located peripherally. In addition to the proposed tumor-related mechanisms of pneumothorax development, a study by Kasahara et al. in animal models showed that long-term therapy with VEGF inhibitors resulted in the distortion of the alveolar structure through the induction of cell apoptosis, indicating that this may contribute to the development of emphysema [35], which is a risk factor for pneumothorax.

Currently, there is also a concomitant conflicting thought process going on in a recent study of patients with breast cancer published by Lodola et al. Their findings indicated that the intracellular Ca^2+^ toolkit is responsible for the pro-angiogenic effect of VEGF and is remodeled in cancer patients and rendered insensitive to VEGF. This finding suggests that VEGF inhibitors including bevacizumab may not have as important a role in tumor vascularization as thought previously and raises the possibility that pneumothorax, in addition to other adverse effects of these agents, may be a result of an alternative effect rather than a direct anti-angiogenic effect. Further in vitro studies that investigate this alternative effect are required [36].

Limitations

First, we only included case reports that might skip authentic primary sources of data that can be solely achieved with clinical trials and observational studies. Second, the association of pneumothorax with bevacizumab is quite rare, thereby we only had a limited sample size of only five patients, thus rendering the power of this study very low. Lastly, not all studies mentioned the ethnicity of the patients, which is an essential factor in evaluating the prevalence in certain regions or groups of ethnicities. It is also possible that the lack of a significant number of case reports describing PTX in association with bevacizumab therapy is due to the lack of widespread knowledge of this association; hence, there may be several unreported such cases. Therefore, it is important to report these cases to spread awareness among clinicians about this potentially life-threatening effect of bevacizumab.

## Conclusions

Based on the above case reports, it can be concluded that although very rare, a clinically significant side effect of pneumothorax can be expected when using chemotherapy regimens containing bevacizumab. Any pulmonary side effect after starting bevacizumab-containing chemotherapy should not be overlooked because it is life-threatening if the diagnosis is delayed. Pneumothorax after bevacizumab therapy can be easily treated if timely diagnosed with a chest tube; hence, clinicians need to keep this differential diagnosis of pneumothorax in their mind when any patient is taking bevacizumab. However, other factors such as concomitant use of cytotoxic drugs in the chemotherapy regimen and radiation therapy used for primary cancers can be involved; therefore, there is a need for further epidemiological studies to establish a causal relationship between bevacizumab and pneumothorax and highlight the mechanisms underlying this effect.
